# Virtual Reality vs. High-Fidelity Mannequin-Based Simulation: A Pilot Randomized Trial Evaluating Learner Performance

**DOI:** 10.7759/cureus.17091

**Published:** 2021-08-11

**Authors:** Maher M Abulfaraj, Justin M Jeffers, Sean Tackett, Todd Chang

**Affiliations:** 1 Pediatric Emergency Medicine, Taibah University School of Medicine, Madinah, SAU; 2 Pediatrics, Johns Hopkins University School of Medicine, Baltimore, USA; 3 Biostatistics, Epidemiology, and Data Management Core, Johns Hopkins Bayview Medical Center, Baltimore, USA; 4 Emergency Medicine, Children's Hospital of Los Angeles, Los Angeles, USA

**Keywords:** virtual reality, simulation, pediatric emergency medicine, learner performance, virtual reality simulation vs. mannequin simulation

## Abstract

Background

Virtual Reality (VR) simulation has been found to be useful in learning technical and non-technical skills. However, empirical data about its efficacy in clinical education are limited. This pilot study compares the efficacy of VR to mannequin-based simulation for learners managing status epilepticus (SE).

Methods

Pediatric and emergency medicine interns at an academic tertiary care referral center were randomized to either VR (intervention, using an Oculus Rift^Ⓡ ^(Occulus from Facebook, Facebook Inc., USA)) or mannequin-based (control) simulation for the same SE scenario. The control group participated in two mannequin-based simulation sessions while the intervention group had a VR session followed by a mannequin-based session. Sessions were one-one with an instructor and held three months apart. Performance was assessed by measuring the time-to-critical actions during the second session.

Results

Of 42 interns, 22 were in the intervention group and 20 in the control group. There was no statistical difference in time-to-critical actions for VR vs. standard groups; for example, VR times (in seconds) compared to standard times were 18.1 (SD 10.5) and 18.9 (SD 15.8) (p=.90) for oral suction, and 61.6 (SD 24.8) and 62.8 (SD 26.9) (p=.82) for IV lorazepam completion.

Conclusion

This pilot trial suggests that VR is feasible for clinical simulation. We did not find a significant difference between the two groups in learner performance. Larger studies are needed to corroborate our findings, investigate the best applications of VR in clinical training, and determine if it could lead to more rapid learning at a lower cost.

## Introduction

Simulation is a popular instructional strategy that has been used in many fields including medical training [1] and applies the concepts of experiential learning [2]. It provides a safe and forgiving environment to practice [1,3,4], which became increasingly important with the restriction of duty hours and growing focus on patient safety [3,5]. This is especially true when it comes to rare but life-threatening conditions [4-6]. There are different modalities for simulation that are used in different settings and for different goals [4,7]. These modalities include task trainers, low and high-fidelity mannequins; however, all of these can be expensive to purchase and require significant resources to operate [2]. It can be used to improve technical and non-technical skills including team performance, communication, and efficiency [2]. There is a growing body of evidence proving its efficacy in medical education [4,8,9]. Studies have shown that simulation increases provider comfort and confidence managing critical situations or performing individual skills [10-16].

Virtual simulation is a newer method that has been gaining popularity in the last decade [17]. Virtual reality (VR) simulation can be designed with a high degree of realism and audiovisual stimulation that can completely seclude participants from their environment allowing for complete engagement [17] and even induce a stress response that can resemble, to some extent, a real-life encounter [18]. VR simulation can improve technical skills [5,19,20] and non-technical skills including teamwork, decision making, and situational awareness [5,17]. VR simulation carries the advantage of being less expensive than mannequin-based simulation, requires fewer resources to implement simulation scenarios, and simulated encounters can be carried out in different settings [5,21,22]. Kayw et al. reported improved post-intervention knowledge and cognitive skills in VR compared to traditional learning methods (lectures, textbooks, or digital sources) [23]. Data comparing the efficacy of VR simulation to mannequin-based simulation when it comes to performance and retention of information is scarce. In a simulated pediatric status epilepticus case, we assessed the feasibility of VR simulation as an effective learning modality and the performance of trainees (and their confidence) when randomly assigned to VR simulation vs a high-fidelity mannequin-based simulation control group.

## Materials and methods

Study design

This was a randomized control trial at an academic tertiary care referral center that receives trainees from different local institutes and programs. It is a level one pediatric trauma and burn care center. Participants included incoming pediatric and emergency medicine interns (academic year 2019-2020) who enrolled voluntarily. All incoming interns were offered the opportunity to participate. All participants had a similar level of experience and had recently graduated medical school. There were no exclusion criteria. Participants were randomly assigned to either a VR simulation group (intervention) or a high-fidelity mannequin-based simulation group (control) with a 1:1 ratio. Time slots were planned to alternate between VR simulation and mannequin-based simulation. Participants who were blinded to the type of simulation planned then selected the time slot that fits their schedule. All participants were proctored by the same instructor (MA) who was a senior pediatric emergency medicine fellow. The first session outlined the objectives of the study, allowed time for orientation, simulation of two cases, an anaphylaxis and status epilepticus followed by debriefing and a questionnaire. The cases were developed to be exactly the same in either VR or mannequin-based simulation. The second session was planned three to four months after the first. This was based on the data reported by Wik et al. that suggested different levels of skill retention at six months depending on the method of training [24]. All participants in the second session completed a case of status epilepticus on a low-fidelity mannequin. The primary outcome was to test trainee performance. It was assessed by extracting the time (in seconds) it took to perform critical actions from the video records of the second session, and a comparison was made between the intervention and control groups. The study was exempt by the institutional review board (IRB00194558). Written consent for participation and video recording was obtained. The manuscript writing process followed the consolidated standards of reporting trials (CONSORT) guidelines [25].

Simulation sessions

First (initial) Session

Participants in the intervention group started their session completing a survey assessing demographics, background in gaming, and subjective confidence in managing status epilepticus. The instructor reviewed the objectives with the participants. These included a stepwise approach to patient evaluation and timely management of recognized issues. Participants were assigned the team leader role whose responsibility is to evaluate the patient and choose the course of action while the nurse and respiratory technician avatars in the VR environment performed actions. The instructor showed screenshots of the VR environment to give a sense of what it looks like and introduced the VR headset and controllers to the participants. Participants were oriented to the VR functions in less than five minutes. This time was based on our experience using the modules/equipment during our regular training sessions. Participants played an orientation module that aimed at explaining how to navigate the simulation cases and perform various actions like examining the patient, choosing airway support equipment, or selecting medications. This module ran between three to five minutes. After orientation, the anaphylaxis case was played, which helped participants get a clear understanding of their role and how to use the various VR functions. After a two-minute break, the participants proceeded to complete the status epilepticus scenario. The debriefing was aimed at exploring participants' feelings and reviewing management guidelines for each case. A post-participation survey was done to assess participant’s satisfaction using VR, cybersickness symptoms, and post-simulation subjective confidence.

The control group sessions were intentionally planned to follow the same structure as the intervention group and allowed the same amount of time for orientation. In these sessions, the instructor performed the actions of the nurse and respiratory technician. Each participant was allowed to explore the room, learn about the various functions of the mannequin, and check equipment availability in the airway, peripheral access, and medication carts. There was no orientation module for this group and the anaphylaxis case was started after participants felt comfortable in the environment. This was followed by the status epilepticus case and debriefing. Participants completed the subjective confidence survey at the end.

Second (assessment) session

Both groups went through a mannequin-based simulation case of status epilepticus as individuals with the instructor. Sessions were video recorded. These records were reviewed to assess the time (in seconds) taken to perform critical actions. Participants were asked to assess their subjective confidence again before and after this session.

Room layout

Control group

**Figure 1:**
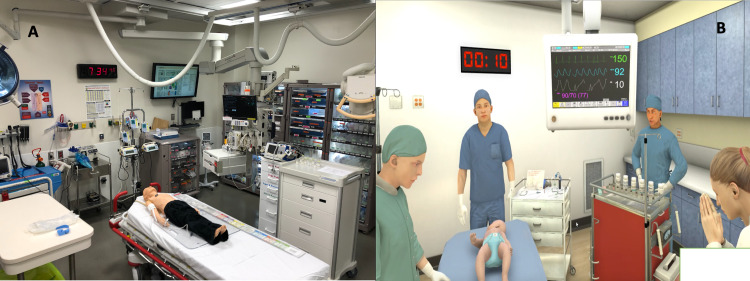
(A) Mannequin simulation room set up in the trauma bay (B) Virtual reality room layout

The simulation was conducted in the pediatric emergency trauma bay. The equipment was intentionally laid out to look as similar as possible to the VR environment. The mannequin was placed on a stretcher and connected to a monitor that was on the patient’s left side along with a list of the medications available to use, which was printed and placed on top of the medication cart on the same side. Airway equipment was placed at the head of the bed. These include a non-rebreather mask, face mask, nasal cannula, bag valve mask, endotracheal tube, laryngoscope, oral and nasal airway adjuncts, and a suction catheter. On the patient’s right side, a peripheral intravenous catheter start kit, blood test vials, and a fluid bag were present. A copy of the vital signs was printed to be used in case of technical issues during the session. The same setting was used for the follow-up sessions for both groups (Figure 1 (A)).

Intervention group

The same trauma bay room was used with the same equipment present; however, the headset and controllers were set on a table on the side. The virtual environment was initially designed to copy the critical care room at the designer’s institution. The participant was placed at the bottom of the stretcher. The vital sign monitor and medication cart were on the patient’s left. The airway cart and respiratory technician avatar were at the head of the bed. The nurse and peripheral venous access tray were on the patient’s right. The instructor was able to observe the participant's performance on the computer screen (Figure 1 (B)).

Equipment

Oculus RiftⓇ headset (Occulus from Facebook, Facebook Inc., USA)

Gaming laptop

Gaumard Super ToryⓇ mannequin (GaumardⓇ Scientific, USA) for the first session

Laerdal MegaCodeⓇ Kid (LaerdalⓇ Medical, Stavanger, Norway) for the second session

A commercially available car seat massager was used to help mimic seizures

GoProⓇ camera (GoPro Inc., USA) was used for recording

Cases

Two cases were developed for the VR (AiSolve Ltd, UK; BioflightVR, USA). The first is a seven-year-old who experiences anaphylaxis with airway involvement. This case would require medical and airway management. The second is a one-year-old in status epilepticus who requires airway management and escalation of anti-epileptic medications. These cases were built under the supervision of two pediatric emergency medicine experts to resemble critical patients in the pediatric emergency room in prior studies [18]. Cases were exactly the same in both the intervention and the control groups. An orientation module was prepared to help users explore the environment and the various functions available.

Debriefing

Debriefing was performed after all sessions following the Promoting Excellence And Reflective Learning in Simulation (PEARLS) framework by Eppich et al. [26]. After exploring the participant’s feelings and concerns, the discussion focused on a stepwise approach for patient’s evaluation and the steps required for stabilization of the airway, breathing, and circulation with emphasis on addressing any issues before moving on to the next step: for example, evaluating and supporting the airway before requesting peripheral vascular access. This was followed by a detailed review of the management guidelines for anaphylaxis and status epilepticus.

Data collection and analysis

Time (in seconds) to completion of certain critical actions was measured to assess differences between the two groups. These actions were chosen based on the major points focused on during the debriefing of the initial session. These include the rapid assessment and management of acutely ill patients according to the pediatric advanced life support (PALS) guidelines following the ABCs and the concomitant medical management of seizures (airway assessment and intervention, e.g., suction, oxygen via non-rebreather or other delivery devices, establishing intravenous access, ordering an antiepileptic medication, and specifically ordering IV lorazepam). The time was assessed by reviewing the video records of the second session. Since one of the goals was to help the participants develop a stepwise approach, the instructor redirected participants to the PALS algorithm if an issue was not addressed promptly. For example, establishing peripheral vascular access would not be accomplished if the airway was not stabilized.

The initial survey included demographics (gender, residency program, video gaming background, and VR experience) and subjective confidence on managing a patient in status epilepticus. The intervention group completed a section on cybersickness symptom checks, including fatigue, discomfort, sweating, dizziness, headache, eye strain, blurry vision, nausea, and upset stomach. All participants completed a survey on subjective confidence after the initial session. The intervention group completed an extra section on cybersickness symptoms and system usability scale using (which was modified from the work of Bangor et al. 2009 [27]).

Confounding factors that could affect the management skills, including a background in neurology and exposure to seizure cases during the period between sessions, were assessed prior to the second session in addition to the reported subjective confidence before and after the session.

Statistical analysis

Descriptive analyses were performed to compare demographics, clinical, gaming experience, and user satisfaction. Time-to-critical actions and subjective confidence were reported as means and standard deviations. Time-to-critical actions were log-transformed before t-tests were applied to test between intervention and control groups. All data were analyzed using Stata (Strata Statistical Software: Release 13, StataCorp, USA)

## Results

Demographics

**Figure 2:**
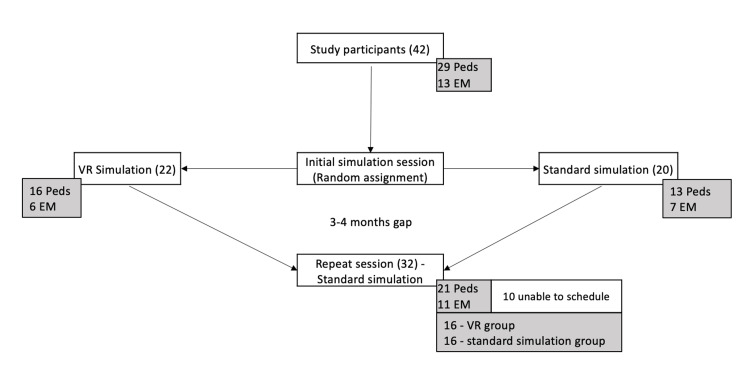
Study design EM: Emergency medicine; Peds: Pediatrics; VR: Virtual reality

Of the 44 interns, 42 participated in the first session while two were not able to schedule. Of these, 31% (13/42) were from emergency medicine and 69% (29/42) from pediatrics. Only one had previous experience with VR gaming. During the second session, 76.2% (32/42) of the total participants were able to participate. The remaining 10 were unable to participate due to time constraints (Figure 2). Among the participants in the second session, 15.6% (5/32) had previous EMS experience, 3.1% (1/32) worked as a nurse, and one had a background in neurology (Table 1).

**Table 1:** Demographic data VR: Virtual reality

		All (n=42)		Standard Simulation (n=20)		VR Simulation (n=22)		p value - No VR vs VR (chi2)
									Frequency	Percentage (%)	Frequency	Percentage (%)	Frequency	Percentage (%)
Gender	Female	25	59.5	11	55.0	14	63.6		0.5
	Male	17	40.5	9	45.0	8	36.4	
Program	Pediatrics	29	69	13	65	16	72.7	0.7
	EM	13	31.0	7	35.0	6	27.3	
Do you play video games?	Yes	16	38.1	8	40.0	8	36.4	0.8
	No	26	61.9	12	60.0	14	63.6	
If you play video games (n=16), how often?	Daily	2	12.5	1	12.5	1	12.5	1.0
	Weekly	6	37.5	3	37.5	3	37.5	
	Monthly	8	50.0	4	50.0	4	50.0	
If you play video games (n=16), do you play VR games?	No	15	93.8	7	87.5	8	100.0	0.3

Time-to-critical actions

**Figure 3:**
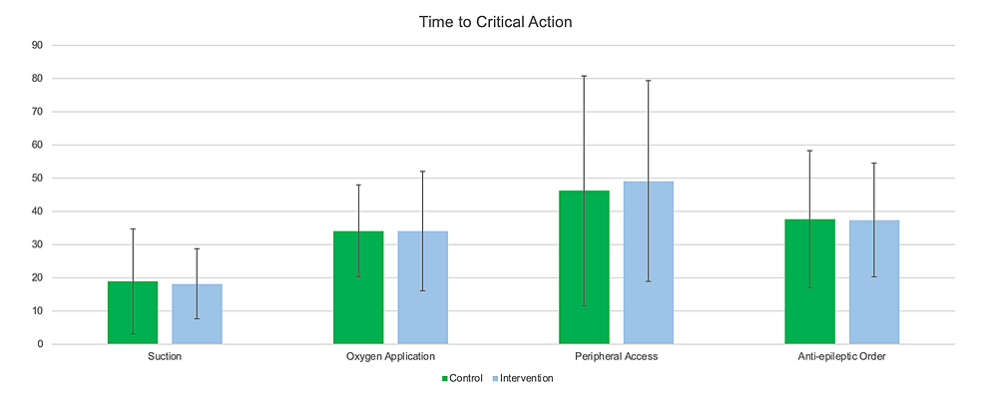
Time-to-critical action between intervention and control groups

Time to clearing the airway secretions with suction was 18.1 seconds (SD 10.5) and 18.9 seconds (SD 15.8) for the intervention group and control group respectively (adjusted p-value 0.9), and administering oxygen with a non-rebreather mask was ordered at 34.1 seconds (SD 17.9), and 34.1 seconds (SD 13.9) for the intervention group and control group respectively (adjusted p-value 0.75) (Table 2). When the entire group was pooled together, the time to clearing the airway with suction was 18.5 seconds (SD 13.2), and oxygen via non-rebreather was at 34.1 seconds (SD 15.8). (Figure 3).

**Table 2:** Time-to-critical actions: comparison of performance between VR and mannequin simulation groups AED: Automated external defibrillators; NRB: Non-rebreather mask

	All (n=32)					Standard Simulation (n=16)					VR Simulation (n=16)					p values
	Obs	Mean	Std. Dev.	Min	Max	Obs	Mean	Std. Dev.	Min	Max	Obs	Mean	Std. Dev.	Min	Max	
Oral suction	32	18.5	13.2	3	63	16	18.9	15.8	3	63	16	18.1	10.5	4	39	0.9
Time to NRB	32	34.1	15.8	10	81	16	34.1	13.9	17	69	16	34.1	17.9	10	81	0.7
Time to O2 completion (Suction and NRB)	32	35.6	16.1	10	81	16	36.2	15.4	20	69	16	35.1	17.3	10	81	0.7
IV order	32	47.6	32.0	4	150	16	46.2	34.6	4	150	16	49.1	30.2	4	119	0.8
Time to first AED order	32	37.5	18.7	1	74	16	37.6	20.6	1	74	16	37.4	17.1	8	66	0.6
Time to IV lorazepam completion	32	62.2	25.5	26	150	16	62.8	26.9	36	150	16	61.6	24.8	26	127	0.8

Subjective confidence

The mean subjective confidence was increased by two points for the intervention group and 1.8 points for the control group (Table 3).

**Table 3:** Subjective confidence: reported confidence before and after each session Std. Dev.: Standard deviation; VR: Virtual reality

		I feel confident recognizing and providing initial management to patients in status epilepticus 1-5												
		All Participants (42)				Standard Simulation (20)				VR Simulation (22)				p values
		Mean	Std. Dev.	Min	Max	Mean	Std. Dev.	Min	Max	Mean	Std. Dev.	Min	Max	
Initial session	Before	2.2	0.7	1	3	2.3	0.7	1	3	2.2	0.7	1	3	0.6
	After	4.1	0.7	2	5	4.1	0.8	2	5	4.2	0.7	3	5	0.8
		All Participants (32)				Standard Simulation (16)				VR Simulation (16)				
Repeat session	Before	2.6	0.6	1	4	2.5	0.6	1	3	2.7	0.6	2	4	0.3
	After	3.7	0.7	2	5	3.6	0.6	3	5	3.7	0.9	2	5	0.8

Cybersickness and system user satisfaction

The VR group included 22 participants. No one reported any symptoms before the VR session. Only one (4.5%) reported mild discomfort and dizziness after completing the VR session, which reportedly resolved without any intervention by the end of the debriefing. Overall, participants rated the user-friendliness of the product as good with a mean of 5.4 out of seven (SD 0.8) (Table 4).

**Table 4:** System usability scale VR: Virtual reality; Std. Dev.: Standard deviation

	1 Strongly disagree - 5 strongly agree					
	Mean	Std. Dev.	Min	Max		
I think that I would like to use this VR simulation frequently	4.1	1.0	1	5		
I thought the VR simulation was easy to use	3.5	1.0	1	5		
I found the various functions in the VR were well-integrated	3.9	1.1	2	5		
I imagine that most people would learn to use this VR simulation very quickly	4.0	1.0	1	5		
I felt very confident using the VR	3.3	1.1	1	5		
Reverse coded items (1 strongly agree - 5 strongly disagree)						
I found the VR simulation unnecessarily complex	4.2	0.5	3	5		
I think that I would need the support of a technical person to be able to use this product	4.0	0.7	3	5		
I thought there was too much inconsistency in the VR simulation	4.3	0.6	3	5		
I found the VR very awkward to use	4.2	0.7	3	5		
I needed to learn a lot of things before I could get going with the VR	4.2	0.7	3	5		
Overall: 1 worst imaginable, 2 awful, 3 poor, 4 ok, 5 good, 6 excellent, 7 best imaginable						
Overall, I would rate the user-friendliness of this product as	5.4	0.8	3	6		

## Discussion

This is a pilot study investigating the efficacy of VR simulation in medical training by comparing it to high-fidelity mannequin-based simulation. Furthermore, it looked into how the use of VR was perceived and assessed the incidence of cybersickness. Our data show that VR simulation has comparable efficacy as high-fidelity mannequin-based simulation. Both groups in our study had similar performance results and there was no statistical difference in the time required to perform critical actions between them. Additionally, participants in the VR group reported high satisfaction rates when using the VR system and equipment. Their incidence of cybersickness was very low.

The cases used in the study aimed to encourage participants to approach critical patients systematically. When the time-to-critical action was assessed, participants in both groups had similar results. This shows that VR simulation can at least produce similar effects on learning.

When compared to mannequin-based simulation, VR is relatively less expensive, requires less human resources to perform, and can be carried out in any setting [5,17,21,22]. We were able to perform VR simulations at any time and location based on the resident's availability. Mannequin-based simulations had to be carefully planned and there was less flexibility when performed. Furthermore, we had a high overall satisfaction score for VR showing it was easy to learn and use, which in turn could increase the intention to use it more if available.

There is reported data about the risk of cybersickness with using VR technology [28]. Our study shows a very low risk of developing such symptoms when using modules built for medical training. Only one person reported mild discomfort and dizziness after finishing the modules. These symptoms were reportedly resolved soon after during the debriefing process. Multiple factors play a role in the risk of cybersickness, including personal and technology-related factors. These could be taken into consideration when planning to decrease the risks.

Both VR and mannequin-based simulation technologies have different characteristics and advantages. As they continue to evolve, we think VR should be used as a method to complement mannequin-based simulation. Future studies can explore the efficacy of VR simulation using robust and standardized testing and assessment methods. More studies should attempt to characterize the best uses for VR simulation and how it can be best incorporated in medical training. This includes the type of cases used and the goals that need to be achieved from simulation. The effects of independent use of VR simulation by learners without supervision is an area that needs exploration. Furthermore, the effects of using both VR and mannequin-based simulation in a longitudinal curriculum should be investigated. Debriefing is an important part of simulation training [29,30]; VR simulation allows for various debriefing methods. We performed in-person debriefing one-on-one with the instructor. Participants also received computer-generated performance feedback. Exploring the appropriate debriefing methods that could be used with VR technology and the usefulness of the computer-generated feedback should also be considered.

Our main limitation was the sample size. This was a convenience sample of incoming trainees at a single institution using specific cases. More robust studies evaluating VR simulation are needed with larger sample size and different scenarios. Attempting to prove equivalency using a non-inferior methodology would require a significantly larger sample size. Our sample size allowed us to explore if a difference may be present. Larger studies will be needed to provide evidence. Our learners came from different backgrounds. Given the randomization of participants into both groups, it was assumed that both groups are equivalent, and no pre-assessment tests were utilized. Post-assessment tests were not utilized as well given that all participants were involved in a standardized one-on-one debriefing covering all learning points. Some have seen and actively managed patients with seizures before or during the period of the study. This may have affected their performance in the assessment session. Debriefing was performed on a one-on-one basis. Although all the focus was on one subject, the absence of a team nature and support could be anxiety-provoking to learners.

## Conclusions

Virtual reality is a feasible method for clinical simulation and training. Although we did not find a significant difference in learning between VR and mannequin-based simulation, future studies should further evaluate the learning outcomes, explore the utility of VR simulation, its application in clinical training, and its effects on learning.

## References

[1] Jeffers J, Eppich W, Trainor J, Mobley B, Adler M (2016). Development and evaluation of a learning intervention targeting first-year resident defibrillation skills. Pediatr Emerg Care.

[2] Zigmont JJ, Kappus LJ, Sudikoff SN (2011). Theoretical foundations of learning through simulation. Semin Perinatol.

[3] Lopreiato JO, Sawyer T (2015). Simulation-based medical education in pediatrics. Acad Pediatr.

[4] Okuda Y, Bryson EO, DeMaria S Jr, Jacobson L, Quinones J, Shen B, Levine AI (2009). The utility of simulation in medical education: what is the evidence?. Mt Sinai J Med.

[5] McGrath JL, Taekman JM, Dev P (2018). Using virtual reality simulation environments to assess competence for emergency medicine learners. Acad Emerg Med.

[6] Louie MC, Chang TP, Grundmeier RW (2018). Recent advances in technology and its applications to pediatric emergency care. Pediatr Clin North Am.

[7] Dull KE, Bachur RG (2012). Simulation in the pediatric emergency department. Clin Pediatr (Phila).

[8] Moya R P, Ruz A M, Parraguez L E, Carreño E V, Rodríguez C AM, Froes M P (2017). Simulation in medical education from the perspective of patients safety [Article in Spanish]. Rev Med Chil.

[9] Motola I, Devine LA, Chung HS, Sullivan JE, Issenberg SB (2013). Simulation in healthcare education: a best evidence practical guide. AMEE guide no 82. Med Teach.

[10] McLaughlin CM, Wieck MM, Barin EN (2018). Impact of simulation-based training on perceived provider confidence in acute multidisciplinary pediatric trauma resuscitation. Pediatr Surg Int.

[11] Pascual JL, Holena DN, Vella MA (2011). Short simulation training improves objective skills in established advanced practitioners managing emergencies on the ward and surgical intensive care unit. J Trauma.

[12] Thomas C, Mackey E (2012). Influence of a clinical simulation elective on baccalaureate nursing student clinical confidence. J Nurs Educ.

[13] Holcomb JB, Dumire RD, Crommett JW (2002). Evaluation of trauma team performance using an advanced human patient simulator for resuscitation training. J Trauma.

[14] Falcone RA Jr, Daugherty M, Schweer L, Patterson M, Brown RL, Garcia VF (2008). Multidisciplinary pediatric trauma team training using high-fidelity trauma simulation. J Pediatr Surg.

[15] van Schaik SM, Plant J, Diane S, Tsang L, O'Sullivan P (2011). Interprofessional team training in pediatric resuscitation: a low-cost, in situ simulation program that enhances self-efficacy among participants. Clin Pediatr (Phila).

[16] Patterson MD, Geis GL, LeMaster T, Wears RL (2013). Impact of multidisciplinary simulation-based training on patient safety in a paediatric emergency department. BMJ Qual Saf.

[17] Bracq MS, Michinov E, Jannin P (2019). Virtual reality simulation in nontechnical skills training for healthcare professionals: a systematic review. Simul Healthc.

[18] Chang TP, Beshay Y, Hollinger T, Sherman JM (2019). Comparisons of stress physiology of providers in real-life resuscitations and virtual reality-simulated resuscitations. Simul Healthc.

[19] Nagendran M, Gurusamy KS, Aggarwal R, Loizidou M, Davidson BR (2013). Virtual reality training for surgical trainees in laparoscopic surgery. Cochrane Database Syst Rev.

[20] Mahmood T, Scaffidi MA, Khan R, Grover SC (2018). Virtual reality simulation in endoscopy training: current evidence and future directions. World J Gastroenterol.

[21] Chang T, Weiner D (2016). Screen-based simulation and virtual reality for pediatric emergency medicine. Clin Pediatr Emerg Med.

[22] Nas J, Thannhauser J, Vart P (2020). Effect of face-to-face vs virtual reality training on cardiopulmonary resuscitation quality: a randomized clinical trial. JAMA Cardiol.

[23] Kyaw BM, Saxena N, Posadzki P (2019). Virtual reality for health professions education: systematic review and meta-analysis by the digital health education collaboration. J Med Internet Res.

[24] Wik L, Myklebust H, Auestad BH, Steen PA (2002). Retention of basic life support skills 6 months after training with an automated voice advisory manikin system without instructor involvement. Resuscitation.

[25] Cheng A, Kessler D, Mackinnon R (2016). Reporting guidelines for health care simulation research: extensions to the CONSORT and STROBE statements. Simul Healthc.

[26] Eppich W, Cheng A (2015). Promoting excellence and reflective learning in simulation (PEARLS): development and rationale for a blended approach to health care simulation debriefing. Simul Healthc.

[27] Bangor A, Kotum P, Miller J (2008). Determining what individual SUS scores mean: adding an adjective rating scale. J Usability Stud.

[28] Stanney KM, Kennedy RS, Drexler JM (1997). Cybersickness is not simulator sickness. Proc Hum Factors Ergon Soc Annu Meet.

[29] Issenberg SB, McGaghie WC, Petrusa ER, Lee Gordon D, Scalese RJ (2005). Features and uses of high-fidelity medical simulations that lead to effective learning: a BEME systematic review. Med Teach.

[30] Sawyer T, Eppich W, Brett-Fleegler M, Grant V, Cheng A (2016). More than one way to debrief: a critical review of healthcare simulation debriefing methods. Simul Healthc.

